# Rupture of Small Intestine and Bladder, from Apparently Inadequate External Violence

**Published:** 1885-08

**Authors:** R. P. Huger

**Affiliations:** Anniston, Ala.


### RUPTURE OF SMALL INTESTINE AND BLADDER FROM APPARENTLY INADEQUATE EXTERNAL VIOLENCE.

BY R. P. HUGER, M. D., ANNISTON, ALA.

As wounds of the abdominal viscera are always of much surgical interest, I report the following case as especially worthy of note, on account of the apparent disproportion between the amount of force exercised and the extent of injury resulting therefrom. It may also serve as a warning to those addicted to athletic sports that violent muscular efforts upon the part of grown men are by no means devoid of danger, and are sometimes attended by consequences which cause lasting regret. Since the occurrence of this case, I have been informed of another very similar to it, in which the father of a large family engaged in a wrestling match, was thrown, suffered intense abdominal pain, threw up large quantities of blood, and died in the course of three days. It is not my intention, however, to lecture young gymnasts, but simply to make the following report:

Charles Crook, a native of Germany, and by occupation a coachman, a man of medium size, and in good health, with the exception of a double inguinal hernia, wrestled with a friend on the evening of October 18th, at about seven o'clock. In a short time he was thrown, and in the scuffle his friend, who was a young man of eighteen years, rather undersized, and weighing 135 pounds, fell upon him in such a manner as to strike the deceased upon the abdomen with his hip. After getting up Crook complained of pain in the bowels. In the course of a few minutes, he walked into a store, about one hundred yards distant, with his hands upon his abdomen, and expressed himself as suffering intense pain. In a short time he became nauseated, and made frequent, but ineffectual attempts to vomit; he was given a dose of morphine, and at 10 p. m. was carried home. During the night he suffered intensely, vomited every few minutes, and at 4 a. m. summoned his physician, Dr. Davis. The doctor found him as related above—abdomen exceedingly tender ; pulse extremely weak; extremities cold. Both herniæ were down. He complained of pain, especially in the region of the bladder. The obvious indications were met; the herniæ were reduced; a catheter passed, and a little bloody urine drawn off. These procedures did not relieve him materially, and hypodermics of morphia were administered.

At 9 a. m., October 19th, I was requested to see him in consultation, and found him in the following condition: Patient in extreme collapse; mind clear; suffering great; pulse barely perceptible; breathing thoracic and labored; countenance expressive of great anxiety; surface of body quite cold and cyanotic; abdomen distended and exquisitely sensitive; stomach extremely irritable.

TREATMENT.

Morphia hypodermically as often, and in such doses as may be necessary to relieve suffering; hot fomentations to abdomen; stimulants, and warm external applications.

*1 p. m—Same Day.*—No appreciable change; relieved to some extent by the morphia, and says he feels a little better.

At 3 p. m. he began to vomit blood in large quantities, and continued to do so until his death, which occurred at 6 p. m.; mind clear to the end.

*Autopsy—7 a. m., October 20.*—Body well nourished; no indications of external violence. Upon section of the abdomen the peritoneum showed evidences of recent inflammation.

The omentum was found partially adherent to the abdominal parietes, and the intestines highly congested, and with adhesive lymph and pus deposited upon its surface. The intestines congested, matted together and bathed in pus at various points; fecal matter was also observed upon them. A rupture was found in the small intestine—ileum—of sufficient size to readily admit the index finger. The bladder was found to have suffered a considerable rupture in its anterior wall, and was almost entirely empty. The abdominal cavity contained a quantity of fluid, composed of the urine which had escaped from the bladder, and also in part probably of serous effusion.

This case having occurred only a few days after reading the very interesting report of Dr. Grindon of "excision of a piece of the intestine," I was deeply impressed as to what would be the duty of a surgeon when confronted with a wound of the abdomen, in which there was no injury of the abdominal parietes, but in which he had every reason to believe there was severe intestinal laceration, and the patient had sufficiently rallied from the shock to justify operative interference, should such be deemed advisable. Should he be content to order the usual treatment in such cases, and despairingly await results, or do recent brilliant avariotomies and the present trend of professional opinion in regard to abdominal surgery, teach another lesson and justify more hope? It seems equally as unphilosophical to treat a wounded intestine with opium and fomentations as to tampon the vagina for a concealed postpartum hemorrhage. I think it unfortunate for mankind that this part of the human anatomy should be regarded as the holy of holies, and many lives probably have been sacrificed to surgical timidity. What then should be the rule of practice in regard to wounds of this region? I can only suggest laparotomy, and after careful searching for the injured part, stitch the edges together, or boldly remove a section of the gut, and thoroughly cleanse the cavity, with antiseptic precautions, should such be preferred. Many probably will agree that such practice would be rational if we certainly knew that such a wound existed. But reason, I think, justifies a step in advance of this—namely, that if an experienced and skillful surgeon felt confident, from the symptoms of the case, that the intestines had suffered injury, he would be warranted in making abdominal section, and satisfy himself upon this point. If, happily, he should have correctly diagnosed his case, he will be in position to probably save the life of his patient. If, on the contrary, no injury should be discovered, he has certainly incurred an additional, but I am inclined to believe, a not unjustifiable risk, and one, too, certainly not as dreadful as we have been accustomed to regard it. The experience of the ovariotomists would be a great comfort to him under such circumstances.

On page 376 of Prof. Bransby B. Cooper's "Lectures on the

Principles and Practice of Surgery," we read: "The symptoms arising from rupture of an intestine without lesion of the abdominal parietes might lead to the supposition that the diagnosis in such a case would be somewhat difficult. Such is not, however, the fact, as there are always sufficiently characteristic points to enable the surgeon to judge accurately of the nature of the injury, and to decide with confidence the proper mode of treatm ent. In this kind of injury, as well as penetrating wounds, of the intestine, collapse is the immedate effect. * * *"

Be this as it may, a fearful mortality certainly attends this class of wounds, and a change of practice in regard to their treatment seems desirable, even though such change should *a priori* appear excessively bold and radical. Time and antisepticism, we sincerely hope, have a valuable revelation for us in connection with this subject.

